# Contribution of Computed Tomography Scan to the Diagnosis of Spinal Tuberculosis in 14 Cases in Assaba, Mauritania

**DOI:** 10.1155/2019/7298301

**Published:** 2019-05-02

**Authors:** Boushab Mohamed Boushab, Noukhoum Kone, Leonardo K. Basco

**Affiliations:** ^1^Department of Internal Medicine and Infectious Diseases, Kiffa Regional Hospital, Assaba, Mauritania; ^2^Department of Neurosurgery, Kiffa Regional Hospital, Assaba, Mauritania; ^3^Aix-Marseille Univ, IRD, AP-HM, SSA, VITROME, Marseille, France; ^4^IHU-Méditerranée Infection, Marseille, France

## Abstract

**Background:**

The incidence of tuberculosis has increased in recent years in both developed and developing countries.

**Objective:**

This retrospective study aimed to review all cases of spinal tuberculosis diagnosed at the Department of Internal Medicine and Infectious Diseases in Kiffa Regional Hospital and assess the role of computed tomography (CT) scan in establishing definite diagnosis.

**Patients and Methods:**

Data were collected from clinical records of patients admitted to the hospital for rachialgia between August 2016 and July 2018.

**Results:**

Fourteen (12.2%) adults with spinal tuberculosis were found among 115 patients with all forms of tuberculosis during the study period. The mean (± standard deviation) age of our patients was 47.5 ± 22.0 years old with male:female (8/6) sex ratio of 1.3. The mean duration of evolution of the disease was 15 months. The presenting signs and symptoms included rachialgia in most patients (93%), associated with segmental spinal stiffness (50%) and/or neurological complications (50%). Diagnosis was established on the basis of clinical history, clinical examination, standard vertebral column radiography, and CT scan. Vertebral imaging showed a clear predominance of lumbar lesions (57%), followed by dorsal (36%) and cervical (7%) involvement. The evolution under treatment was favorable, with the exception of two cases of medullary compression.

**Conclusion:**

Spinal tuberculosis is the most common form of osteoarticular tuberculosis. It affects predominantly lumbar and dorsal vertebrae. In the absence of histological confirmation, the presence of back pain associated with major radiological signs of spondylosis disc disease seems to justify the use of CT scan to confirm the diagnosis of this pathology.

## 1. Introduction

Tuberculosis (TB) is a public health problem worldwide [1]. Spinal tuberculosis, also known as “Pott's disease,” is one of the complications affecting about 1 to 2% of all cases of tuberculosis [2–4]. The coexistence of human immunodeficiency virus (HIV), which is endemic in many regions where TB is commonly encountered, adds to the burden and complexity of clinical management [2]. Among different forms of skeletal TB, the spine is most common affected site [5–7], followed by the hip and knees. The exact incidence and prevalence of spinal tuberculosis in most parts of the world are unknown. In countries with a high burden of pulmonary TB, the incidence of spinal TB is expected to be proportionately high. Spinal TB is a serious complication due to the possibility of permanent neurological damage [7].

Computed tomography (CT) scan has become a major contributor to confirm diagnosis, assess the degree of injury, and search for complications of spinal TB [8]. There is no previous study on the description of clinical aspects of spinal TB in Mauritania. The aims of the present study were to determine the prevalence of spinal TB, describe typical radiological aspects found in our patients, and evaluate the importance of CT scan in establishing a reliable diagnosis of spinal TB in a Mauritanian setting.

## 2. Materials and Methods

This is a retrospective analysis of 115 TB patients admitted in the Department of Internal Medicine and Infectious Diseases of Kiffa Regional Hospital over a period of 2 years (August 2016 - July 2018). The diagnosis of spinal TB was based on a set of clinical, radiological, and therapeutic observations (i.e., a satisfactory evolution under antituberculosis treatment). Diagnosis was not established on the basis of bacteriological or histological examinations due to the lack of a technical platform. However, vertebral CT scan was performed in all patients with suspected spinal TB. Quadruple antituberculosis treatment administered to all patients consisted of the combination regimen: rifampicin (10 mg/kg/day), isoniazid (5 mg/kg/day), ethambutol (20 mg/kg/day), and pyrazinamide (30 mg/kg/day), for two months, followed by dual therapy with rifampicin and isoniazid at the same doses for 10 months. Treatment was started in all patients with radiological confirmation of diagnosis of spinal TB. A favorable response to the treatment was defined as the recovery of the body weight, disappearance of pain and fever, and evidence of radiological improvement. Each patient was seen at least 2 times after completing the full anti-TB treatment course.

## 3. Results

During the 2-year study period, 14 (12.2%) cases of spinal tuberculosis were diagnosed among 115 patients with all forms of TB. The mean (± standard deviation [SD]) age of 14 patients was 47.5 ± 22.0 years old. The sex ratio (M/F) was 1.3. Many of the patients (8/14, 57%) came from suburban areas, and 6 of 14 (43%) were jobless. Pulmonary or extrapulmonary (but not spinal) TB was diagnosed prior to consultation in our hospital in 5 (36%) patients. Three patients had a concomitant chronic disease: noninsulin-dependent diabetes, rheumatoid arthritis, or gout. The mean (± SD) time to diagnosis from the appearance of first clinical signs attributable to discovertebral infection was 13.8 ± 4.0 months.

**Figure 1:**
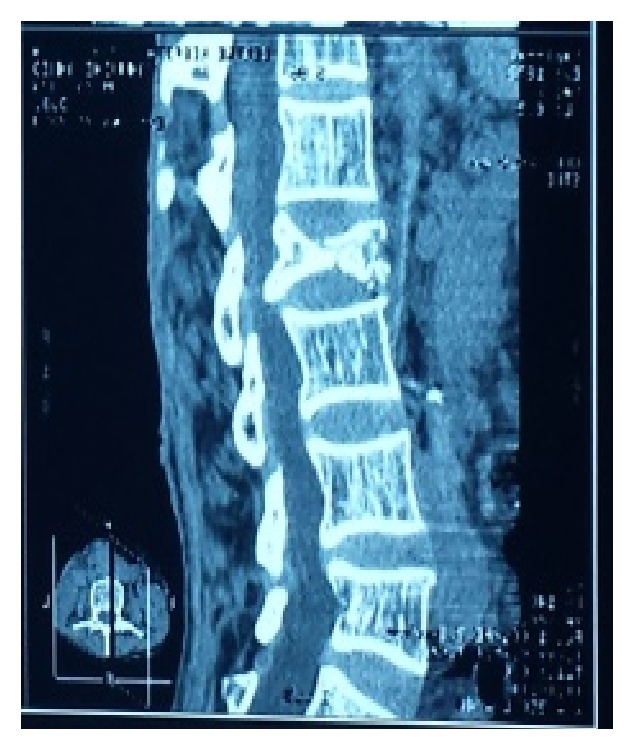
A 45-year-old paraplegic patient with tuberculous spondylitis and osteolysis of L2.

**Figure 2:**
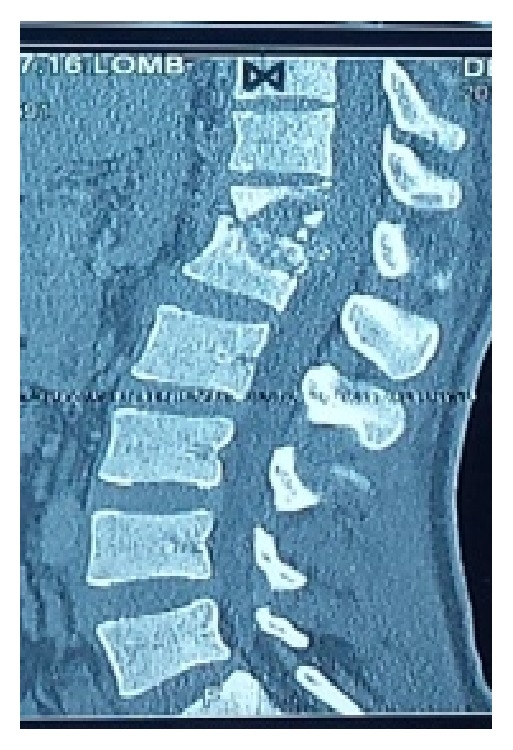
A 60-year-old paraplegic patient with tuberculous spondylitis and osteolysis of D12-L1 and 40° kyphosis.

**Figure 3:**
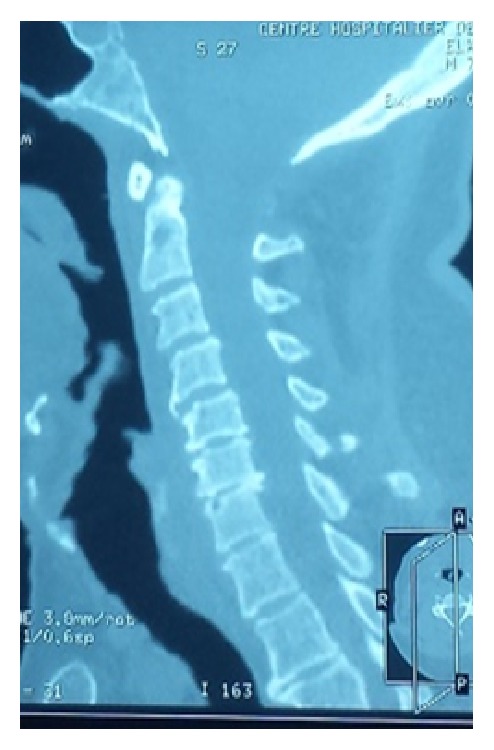
A 57-year-old patient with tuberculous cervical spondylitis and osteophytosis of C4-C5-C6.

The presenting signs and symptoms were inflammatory rachialgia (13/14 patients; 93%) associated with segmental spinal stiffness (7/14; 50%) or deterioration of the general state and signs of spinal compression (7/14; 50%). CT scan was performed in all 14 patients and confirmed the diagnosis of spinal TB. It showed perilesional osteocondensation (14/14; 100%) and sequestrum (5/14; 36%). According to the topography of the lesions, there was a clear predominance of lumbar lesions (8/14; 57%) (Figure 1), followed by dorsal (5/14; 36%) (Figure 2) and cervical lesions (1/14; 7%) (Figure 3).

A quadruple anti-TB treatment was prescribed for the first two months, followed by isoniazid and rifampicin bitherapy for at least the next 10 months. A corset immobilization was indicated in six patients who were at risk. In two patients who presented with neurological complications related to spinal epiduritis, corticosteroid treatment associated with anti-TB chemotherapy resulted in clinical amelioration. During the clinical course of one patient, abscess developed in thoracic spine D11-L2 but was treated successfully with antibiotics (ciprofloxacin 500 mg twice daily for two months). Spinal TB was cured in this patient.

**Figure 4:**
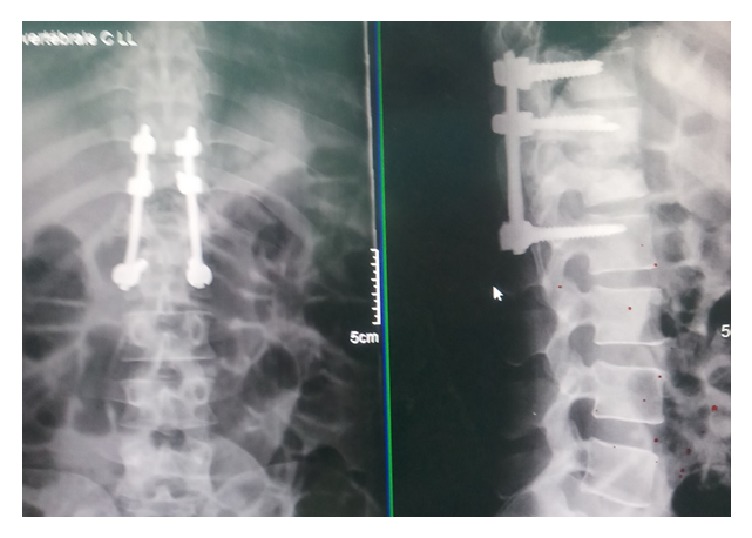
After incision of abscess followed by excision of necrotic tissue, corpectomy and fixation of D11-L2 disc using a plating system were performed.

Of 14 patients with spinal TB included in the present study, 11 (79%) recovered without sequelae after 12 months of treatment, 1 patient required surgical intervention (Figure 4), and 2 patients died (Table 1).

## 4. Discussion

Spinal TB is commonly observed in developing countries where TB is highly prevalent and is also increasingly observed in developed countries [2, 3]. Although skeletal TB occurs mostly in the spine, followed by the hip, knees, and foot, or ankle, the epidemiology of extrapulmonary TB and in particular musculoskeletal TB remains largely unknown in areas of high prevalence of TB [4]. The average age of our patients at the time of diagnosis was 47.5 years, which was similar to other studies conducted in Africa that reported age of adult patients with musculoskeletal TB ranging from 40 to 60 years [7, 9–11]. A slight male predominance of the disease has often been reported in the literature [8, 12]. As noted by other authors, patients with spinal TB were of lower social class [8, 13], and many of them did not present earlier with signs of pulmonary TB [10]. Several risk factors have been reported, including diabetes, nephropathy, and recent spine surgery [7]. In our study, some patients had diabetes, rheumatoid arthritis, or gout.

The clinical presentation, insidious evolution of signs and symptoms, and the absence of prominent general signs are generally responsible for a diagnostic delay ranging from 1 to 26 months [6, 8, 14]. In our series, the average delay was 13.8 months, which was due to the frequent use of traditional medicine for initial therapy, poor access to health facilities, and poverty. The majority of patients were afebrile during the first visit, and routine laboratory examinations were not helpful in establishing the diagnosis. Inflammatory rachialgia with segmental spinal stiffness was the major presenting symptoms. However, the total absence of an inflammatory syndrome does not exclude the diagnosis of spinal TB since 12 to 50% of patients may have no biological signs of inflammation [15]. Half of our patients had neurological complications, similar to the reports from other authors, i.e., 20 to 50% [7, 8, 16]. The neurological deficit depends on the extension of the infectious process, compression, and epidural extension [17].

Standard radiography suggested spinal TB in our patients. However, standard vertebral X-ray may be normal even in the presence of spinal TB [18]. In such cases, CT scan is a valuable diagnostic tool, especially in detecting abscess within the vertebral canal. Bone destruction is well visualized in CT scan. The association of bone lesions with sequestrum and peritoneal abscess containing bone fragments is, in some patients, pathognomonic of TB [7, 19]. The presence of a bone sequestrum, osteosclerosis, and epidural or soft tissue abscess is an important element in orienting the diagnosis [19, 20].

The distribution of lesions along the spinal column observed in our series, with the thoracolumbar area being most affected, has also been reported by other authors in adult patients [6–8, 21, 22]. This finding is explained by the spread of TB from the initial pulmonary focus via vertebral, intercostal, and lumbar arteries [23]. Cervical involvement is rare, accounting for about 5% of spinal TB [7, 24]. Cervical involvement was observed in one patient in our study. Usually, pain associated with spinal TB affects 2 vertebral segments: cervicolumbar, dorsolumbar, or cervicodorsal segments [25]. Concomitant involvement of all 3 spinal segments is rare, and only 3 cases have so far been reported in the literature [26]. Spinal TB in most of our patients (93%) was characterized by a single location. Other authors have also found a single location in more than 70% of cases [8, 27].

In our study, the diagnosis of spinal TB was made on the basis of clinical and radiological evidences, including CT scan, confirmed by a favorable therapeutic response to anti-TB therapy. However, in developed countries with a modern medical diagnostic laboratory and surgical practice, definitive diagnostic methods of spinal TB are based on bacteriological examinations to detect, cultivate, and isolate* Mycobacterium tuberculosis* if an abscess can be accessed for biopsy or if biopsy is performed preoperatively. These procedures were not performed in our study due to inadequate laboratory facilities. Instead, CT was performed when indicated as it is more sensitive than standard radiography to diagnose spondylodiscitis. In health facilities without sophisticated medical laboratory for bacteriological culture, CT imaging is probably the best available diagnostic method. If available, magnetic resonance imaging is even more accurate and sensitive than CT.

A well-managed anti-TB treatment for a period of 12 to 18 months is usually sufficient to achieve cure. The average duration of treatment of our patients was 12 months. In addition to drug treatment, surgical treatment was necessary in one of our patients. Because there are surgical risks and medical treatment alone may be adequate to cure spinal TB, surgical indications were based on the following: the failure of a well-conducted medical treatment, in which case surgery is the only alternative for the paraplegic patient; paraplegia with significant bone destruction and/or vertebral instability; acute compression due to bulky abscess and fistula or mechanical compression by sequestrum; or paraplegia with kyphosis that requires correction or prevention of aggravation. The rate of surgical intervention (1/14 patients; 7%) in our study was low because this type of surgery is reserved for patients with no other alternatives in the context of very limited resources in our country, including the technical platform, availability of intensive care units, the experience of surgeons and anesthesiologists, medical costs, and the overall risk benefit assessment of the patient [28]. In other studies performed in developing countries, a similar low rate of surgical treatment for spinal TB was observed [5, 7, 9, 13, 23, 25, 28].

The role of surgery in treating spinal TB has been controversial over the past decades, and there is no standard recommended surgical procedure for spinal TB [23]. Although a routine use of surgery is not advocated by most medical practitioners, in case of nerve compression leading to neurological deficit, surgery is definitely beneficial to patients for a more rapid recovery and shorter duration of use of the corset. In our patient, a stable fixation was achieved using screws and an osteosynthesis plate spanning the posterior pedicular D11-L2. Similar successful results were obtained by other authors [13, 18, 25]. A more sophisticated surgical treatment practiced in advanced countries involves a double anterior approach followed by posterior intervention, which provides additional plaster and allows early ambulation. Despite successful surgical operations that may lead to less kyphosis, more rapid pain relief, and bone fusion, until more evidence for immediate and long-term benefits is available after surgical interventions, a more conservative approach based on anti-TB medical treatment and case-to-case assessment for possible advantages of surgical procedures is probably more appropriate in developing countries.

Drug treatment of spinal TB is based on anti-TB treatment which should be maintained for at least 12 months [1, 5, 14, 16, 24, 28–30]. In addition to drug treatment, immobilization can reduce pain and limit sequelae. Six patients in our series had been immobilized, which improved case management in all these cases.

Commonly observed complications of spinal TB include kyphosis, neurological deficit (motor and sensory impairment), spinal deformity, and abscess. The risk of kyphosis can be limited by using braces to protect the spine. Neurological deficit and spinal deformity can be improved and corrected in most cases with effective anti-TB therapy. Likewise, abscess, when present, usually resolves with effective anti-TB therapy.

## 5. Conclusion

Spinal TB is still common in Mauritania. The insidious clinical picture often leads to a delay in diagnosis and complications. Modern imaging techniques, including CT scan, are helpful in confirming the diagnosis of spinal TB. Early diagnosis and prompt and appropriate treatment can considerably improve the prognosis of spinal TB. Moreover, control of TB during its early stages can prevent spinal TB.

## Figures and Tables

**Table 1:** Characteristics of 14 patients with spinal tuberculosis included in our study.

Characteristics	Case 1	Case 2	Case 3	Case 4	Case 5	Case 6	Case 7	Case 8	Case 9	Case 10	Case 11	Case 12	Case 13	Case 14

Age (yr)	12	15	20	60	68	61	45	37	82	26	50	65	65	60
Sex^1^	M	M	M	F	F	F	F	F	M	M	F	F	M	F
Pulmonary TB	Yes	Yes	No	Yes	No	Yes	No	No	No	Yes	No	No	No	No
Concomitant disease				Diabetes	Gout	RA^2^					AH^3^			
Time to diagnosis (months)	12	21	9	18	17	22	13	14	17	12	9	12	9	9
Presenting symptoms														
Spinal stiffness	Yes	Yes	No	No	Yes	No	Yes	Yes	Yes	Yes	No	No	No	No
Rachialgia	Yes	Yes	Yes	No	Yes	Yes	Yes	Yes	Yes	Yes	Yes	Yes	Yes	Yes
Spinal compression	No	No	Yes	No	Yes	Yes	No	No	Yes	Yes	Yes	Yes	No	No
Location of the lesion^4^	D5	L4-L5	D10-D12	L2	D3-D5	D12-L1	L4	L4-L5	L2-L3	L4-L5	C4-C6	L4-L5	L3-L4	D11-L2
Surgical treatment	No	No	No	No	No	No	No	No	No	No	No	No	No	Yes
Corset immobilization	No	No	Yes	No	Yes	No	No	No	Yes	No	Yes	Yes	No	Yes
Outcome	cured	cured	cured	cured	cured	cured	cured	cured	deceased	cured	deceased	cured	cured	Yes

1M, Male; F, Female;

2RA: Rheumatoid Arthritis;

3AH: arterial hypertension;

4D, dorsal vertebrae; L, lumbar vertebrae; C, Cervical vertebrae.

In all 14 patients, spinal TB was confirmed by CT scan, and the standard anti-TB treatment was administered.

## Data Availability

The clinical data used to support the findings of this study are included within the article.
